# RURAL ADULTS' BEHAVIOR CHANGES DURING COVID-19: A LATENT CLASS ANALYSIS

**DOI:** 10.1093/geroni/igac059.1938

**Published:** 2022-12-20

**Authors:** Tim Killian, Betsy Garrison

**Affiliations:** University of Arkansas, Fayetteville, Arkansas, United States; University of Arkansas, Fayetteville, Arkansas, United States

## Abstract

The advent of the SARS-CoV-2 virus necessitated behavior changes for most older adults. Changes were largely associated with voluntary or enforced compliance with social distancing policies. Whereas the value of social distancing for mitigating the spread of the dangerous SARS-CoV-2 virus is not the subject of serious debate among public health advocates, policies that encouraged or enforced social distancing have been met with skepticism in the general public. Understanding patterns in behavior changes may be helpful to public health professionals and policymakers who develop health communication and revised guidelines in the efforts to contain the spread of the SARS-CoV-2 and future public health infections. Our study examined patterns in behavior changes. This study had two aims. First, using survey responses from 661 adults living in rural areas about changes they made to 20 different behaviors, we identified four classes of behavior changes: (1) non-social distancers (10.7%), (2) social distancers (54.0%), (3) social distancers making civic exceptions (24.0%), and (4) social distancers making religious exceptions (11.3%). Second, polytomous logistic regression was used to predict the probability of membership in those latent patterns of behavior change. Predictor variables included demographic variables (i.e., age, sex, and race) and variables theoretically predicted to covary with patterns of behavior change (i.e., social trust, self-assessment of health, experience of pain, and depressive symptoms). This study provides insight into behavior changes during the outbreak of the SARS-CoV-2 virus that may be used by practitioners to understand variations in how people responded to the outbreak.

